# Traffic flow data quality control under video frame rate considering section-level geospatial similarity

**DOI:** 10.1371/journal.pone.0320567

**Published:** 2025-05-06

**Authors:** Yue Chen, Jian Lu

**Affiliations:** 1 Jiangsu Key Laboratory of Urban ITS, Southeast University, Nanjing, China; 2 Jiangsu Province Collaborative Innovation Center of Modern Urban Traffic Technologies, Southeast University, Nanjing, China; 3 School of Transportation, Southeast University, Nanjing, China; 4 School of Civil Engineering and Transportation, Nanchang Hangkong University, Nanchang, China; Southwest Jiaotong University, CHINA

## Abstract

The quality of traffic flow data is very important to the effective management and operation of urban traffic system. At present, most traffic flow data used in traffic flow research come from road sensors, but the shortcomings of long sampling period and sparse sampling points affect the quality control of traffic flow data. To solve these problems, we propose a traffic flow data quality control method under video frame rate considering cross-sectional geospatial similarity. Under this framework, we design a video-based multi-section traffic flow data collection method to improve the availability of spatiotemporal similarity of traffic flow data. Further, combining the advantages of traffic flow data in space-time dimension under video frame rate, a data repair method based on cross-sectional geospatial similarity and piecewise interpolation is proposed, and a multi-sectional combined repair model based on LSTM is constructed. Experiments were carried out on several road cross-sections, and the results show that the proposed model has the best data repair effect under different sampling periods, different missing rates and different missing types, and has certain competitiveness in traffic flow data quality control.

## Introduction

With the rapid development of cities and the rise of car ownership, traffic pollution, traffic congestion, traffic accidents and other problems are increasingly significant, Intelligent Transportation System (ITS) has played an important role in order to solve these urban traffic problems [1]. ITS is a comprehensive application of advanced science and technology in transportation and service control [1]. ITS greatly strengthens the relationship between vehicles, roads and users in geographical space, among which the data quality of traffic flow is the data support of traffic congestion warning, traffic signal control, road speed limit control and other practical applications. Traffic flow data quality control, as the foundation and key component of ITS and even geographic information system, has been widely studied [2].

Traffic flow data is the basis of all traffic flow research, including short-term traffic flow prediction, traffic condition warning, traffic signal timing, and even long-term traffic management, design and planning. At present, the data source of traffic flow research is mostly vehicle detectors. Table 1 lists some relevant studies on short-term prediction of traffic flow data collected by vehicle detectors in recent 10 years, focusing on the sampling period and research objects. From the perspective of sampling period, the sampling period of traffic flow data is mostly 5min, accounting for 65%; the second was 15min, accounting for 23%. Therefore, the sample size based on vehicle detector can be collected in a relatively short period of time, which is not conducive to the quality control of traffic flow data highly dependent on spatiotemporal similarity. Further observing the research objects of traffic, it can be found that the research objects of traffic flow are mostly flow, followed by speed. There are few studies on occupancy and even less studies on traffic density, which is not conducive to the comprehensive grasp of traffic flow state.

**Table 1:** Traffic flow research based on data collected by vehicle detectors.

Author	Year	Sampling period	Research object
Hu *et al*. [3]	2024	5min	flow
Ye *et al*. [4]	2024	5min, 10min, 15min	flow
Wang *et al*. [5]	2024	15s	flow
Luo *et al*. [6]	2024	5min	flow
y Wu *et al*. [7]	2024	5s	flow
Yu *et al*. [8]	2023	5min	flow
y Su *et al*. [9]	2023	5min	flow
y Xu *et al*. [10]	2023	3min	speed
Yang *et al*. [11]	2023	5min, 12min	flow, speed, occupancy
Kumar *et al*. [12]	2023	5min	speed
y Bao *et al*. [13]	2023	5min, 15min	flow, speed, occupancy
Zhuang *et al*. [15]	2023	15min	flow
Li *et al*. [16]	2022	15min	flow
yy Chen *et al*. [17]	2022	5min	flow
Lin *et al*. [18]	2022	5min	flow
Hu *et al*. [19]	2022	5min	speed
Yang *et al*. [20]	2022	5min	speed
Sun *et al*. [21]	2022	5min	flow
Xu *et al*. [22]	2020	5min	flow
y Zhang *et al*. [23]	2019	5min	flow
y Yang *et al*. [24]	2019	5min	flow
Tian *et al*. [25]	2018	15min	flow
y Ma *et al*. [26]	2017	2min	flow
Cai *et al*. [27]	2016	5min	speed
Ma *et al*. [28]	2015	2min	speed
Guo *et al*. [29]	2014	15min	flow

There are two main challenges to achieving efficient and accurate traffic flow data quality control. From the perspective of time, ITS can collect different traffic parameters through sensor devices, but the sampling period is often too long, which makes the traffic flow system in a state of sparse data. From the perspective of space, for a single road cross-section, most sensor devices are only collected in one cross-section, and it is impossible to effectively use the highly correlated data at the cross-section level in a small range.

To this end, traffic flow monitoring video is used to collect traffic flow data in multi-section at video frame rate, and a traffic flow data quality control method is proposed under video frame rate considering cross-section geospatial similarity. The research motivation of this paper is to make full use of the advantages of traffic flow data under video frame rate in time dimension and space dimension, conduct traffic flow data quality analysis, and design traffic flow data quality methods. It includes cross-sectional geospatial similarity analysis, preprocessing of continuous missing, segmental interpolation repair based on cross-sectional geospatial similarity, and multi-sectional combined repair model based on LSTM (Long Short-Term Memory).

The main research contents and contributions of this paper are as follows:

1. A video-based multi-section traffic flow data collection method is designed. The traffic flow data based on video collection can effectively solve the sparse traffic flow data problem in the time and space dimension of the traffic flow system, and effectively improve the availability of spatio-temporal similarity of traffic flow data.
2. A data repair method based on cross-sectional geospatial similarity for continuous missing preprocessing and piecewise interpolation is proposed. The method can efficiently pretreat continuous and random missing and lay a foundation for subsequent accurate repair.
3. A multi-section combined repair model based on LSTM is proposed, which can effectively grasp the strong spatial correlation of traffic flow data on multi-sections and further improve the data quality of traffic flow based on deep learning.

The rest of this paper is organized as follows: Sect 2 provides related research on traffic flow data quality control. Sect 3 introduces the video-based multi-section traffic flow data collection method, the data repair method of continuous missing preprocessing and piecewise interpolation based on cross-section geospatial similarity, and the multi-section combined repair model based on LSTM. In order to further evaluate the effectiveness of data quality control methods, Sect 4 analyzes and compares the data repair efficiency and influencing factors, and describes the data used in this study. Finally, this study is discussed and summarized, and the possible work in the future is prospected, in order to be helpful for the follow-up research.

## Related work

The quality of traffic flow data has a great impact on the short-term prediction of traffic flow. If the raw data is applied to the later data analysis and modeling process, unreliable and even wrong analysis results will appear. Therefore, before analyzing and utilizing the data, traffic flow data quality control is needed to ensure the reliability and accuracy of the data. For this reason, scholars have put forward many different data quality control methods from different angles.

### Identification of wrong data

For the identification of wrong data, scholars generally adopt threshold discrimination and logical discrimination.

1. Threshold discriminant method

The mechanism of threshold discrimination is to define the upper limit and lower limit of traffic flow data, and judge the data greater than the upper limit or less than the lower limit as the error value, so that the traffic flow data is in a reasonable range. According to different threshold conditions, threshold discrimination can be divided into parameter threshold discrimination, threshold discrimination based on road design principles, and threshold discrimination based on traffic conservation law.

In 1976, Payne [30] first proposed to use the single-parameter threshold method to identify traffic wrong data and apply the culling method to process the wrong data. Jacboson *et al*. [31] took single-coil detection data as the research object and proposed an effective rule for traffic flow data: When the time convergence degree is 20 seconds, the single-lane flow cannot exceed 17 pcu; the minimum value range of time occupancy is [0,10%]; Since the single-coil cannot directly collect the speed data, the speed threshold is not given in the rule. Cleghorn *et al*. [32] added threshold conditions such as the flow value must not be negative and not exceed the maximum capacity of the road, the average location speed must not be negative and generally not exceed 120 km/h, based on general road design principles. Nam *et al*. [33] proposed a fault data identification and processing method for locating traffic parameters based on traffic flow conservation law.

On the basis of the basic threshold method, researchers have proposed a more accurate discriminating method. Chen *et al*. [34] judge whether the magnetic induction coil works normally by observing the time-rate series images of the flow and occupancy. The detection process is simple and straightforward, but the defect is that it cannot identify the wrong data at a single point. Vanajakshi *et al*. [35] established a traffic conservation law optimization model by analyzing the continuous traffic flow numbers collected by a series of detectors and taking the minimum sum of squares of accumulated traffic data differences of adjacent detection sections as the objective function. Jain and Coifman [36] designed five threshold test criteria for magnetic induction coil data: (1) In the data collected from 15:00 to 22:00, the amount of zero traffic flow and zero occupancy data exceeds 50% of the total data; (2) Traffic flow is zero and occupancy is non-zero; (3) Traffic flow is non-zero and occupancy is zero; (4) Abnormally high traffic flow or occupancy; (5) For a period of time, the traffic flow and occupancy values do not change, and if any of the above conditions are met, the data can be determined to be abnormal.

2. Logical discrimination

Threshold discriminant method can initially delete abnormal data that is not within the reasonable range, but can not completely solve the problem of identifying wrong data. Therefore, scholars proposed a logical discrimination method based on traffic flow theory: When the value of a certain traffic flow parameter is 0, one of the possibilities is that no traffic flows through the detection cross-section in the period of time, and the other is that there is a fault situation and wrong data is generated. In this case, it is necessary to make logical judgment based on the three-parameter relationship of traffic flow and further eliminate abnormal data.

Lomax and Turner [37] proposed more accurate wrong data judgment criteria based on the traffic flow theory, which mainly include: (1) The parameters show sharp fluctuations in continuous time; (2) For the same cross-section, the detector obtains completely different values; (3) The detectors at different locations collected exactly the same data: (4) The collected data had very low similarity with the historical data of the same period: (5) The traffic flow, speed and occupancy were inconsistent. Based on historical traffic flow data, Park *et al*. [38] screened outliers that did not conform to the overall traffic trend.

### Repair of missing data

Due to sensor failure, communication methods, weather conditions and other factors, traffic data will be affected to varying degrees, and data missing is inevitable. The missing data is always random, which brings great difficulties to data repair. How to deal with missing values is the primary problem in data mining, and the repair accuracy directly affects the reliability of regional traffic flow prediction, traffic safety [39], vehicle network [40] and other related studies. Therefore, it is very important to study the repair method of missing value in traffic data. In the research of missing data repair, according to the classification of data repair principles, data repair methods are usually divided into three categories: Statistics-based data repair method [27], interpolation-based data repair method [41], and prediction-based data repair method [42].

1. Statistics-based method for repairing missing data

The repair of missing data based on statistics is to obtain the characteristic relationship between traffic flow data and repair the missing data by using mathematical statistical relationship and appropriate statistical principle. This method usually consists of two steps: First, assume a probability distribution model of the data; Secondly, based on the observed data, the model parameters are iteratively estimated while the missing data is estimated [43]. Markov Monte Carlo (MCMC) [44] and Probabilistic Principal component analysis (PPCA) [45] are two representative methods in this field. PPCA extends Principal Component Analysis (PCA) [46] by using the expected value maximization algorithm. By treating the missing data as an unobserved hidden variable and using the information from the existing data to infer the possible values of the missing data, the resulting probabilistic model can better handle the missing data [47]. Qu *et al*. [45] used PPCA technology to repair missing data using daily periodicity and interval changes of traffic data, but the model did not consider spatial changes of data, so its accuracy would be affected by spatial scale. If the spatial scale is too large, the model may not be able to capture local changes in the data, resulting in differences in repair results [48].

Methods based on statistical learning usually have strong assumptions about traffic flow data. If the assumed probability distribution captures the changes in traffic flow well, good performance can be obtained. However, the actual traffic flow environment is quite complex, and it is difficult to make reasonable assumptions about the characteristic relationship between traffic flow data according to the statistical principle. In addition, if the sampling frequency of traffic flow parameters is low, the rationality of the hypothesis will be weakened.

2. Interpolation-based method for repairing missing data

Interpolation based missing data repair is to make use of the relationship between missing data and adjacent data to repair missing data [51]. Commonly used interpolation methods mainly include mean value method, median method, regression algorithm, time series [49] and so on. For example, Zhong *et al*. [50] compared and analyzed the repair effect of genetic neural network regression model, factor model and ARIMA model based on different scenarios, and concluded that the repair effect of genetic neural network regression model was better than that of factor model and ARIMA model. Another example is Zhang [52], based on K-nearest neighbor algorithm and gray distance, the grey K-nearest neighbor data missing complement model is established.

The mean method and median method respectively take the mean and median of the adjacent data of the missing data as the repair data, which are only applicable to the case of smooth fluctuation of traffic data [53]. Linear regression is easy to apply, but it does not consider the temporal and spatial characteristics of traffic data, and lacks stability, and the time series cannot fill in the information of the time point after the missing value [54]. Autoregressive Integrated Moving Average (ARIMA) [55, 56] and seasonal ARIMA models [57, 58] are representatives of auto-regressive missing time series data repair algorithms. These models take into account the autocorrelation and seasonality in time series data, can capture the time dynamic characteristics of data, and process nonlinear data to a certain extent, but the effect is not ideal for highly nonlinear data.

The matrix decomposition method can extract potential data features from the original data, so as to better understand the structure and pattern of the data, so the application of matrix decomposition and related tools is becoming more and more common in the field of traffic data repair. Low-rank matrix factorization [59], as a typical method, achieves efficient processing of large-scale data by compressing the data into a lower dimensional subspace for representation. Tan *et al*. [60] introduced the matrix extended tensor model into traffic data modeling for the first time and proposed an advanced interpolation method based on tensor decomposition. Luo *et al*. [61] proposed a traffic data filling method based on improved low-rank matrix decomposition, where information on missing data is reflected into the coefficient matrix, and spatio-temporal correlation properties are applied to obtain more accurate filling results. Su *et al*. [62] proposed the road distance adjacency matrix to represent the spatial information of the road network, which can be combined with the original traffic data to complete the missing traffic data in the merged traffic data. Other matrix decomposition techniques reconstruct traffic flow data into a three-dimensional tensor [63], and apply matrix decomposition methods in model training, researchers use Bayesian statistics [64], extend the existing tensor decomposition method [65, 66], or use additional preprocessing algorithms [67] to optimize the model. However, the computation of high-dimensional tensors requires high memory and computational power, and if new sparse tensors are entered, the network needs to be retrained, resulting in serious time complexity problems. In addition, this type of repair model does not take into account the road network topology, resulting in a lack of scalability and difficulty in providing stable repair in complex missing cases.

Overall, interpolation-based missing data repair is highly dependent on the spatio-temporal correlation of traffic flow parameters. When the sampling frequency is low or there are few nearby detection points, the sparse data will increase the difficulty of accurately grasp the spatio-temporal correlation of traffic flow parameters, resulting in greatly reduced repair effect.

3. Prediction-based method for repairing missing data

Prediction-based repair of missing data is to take the missing data in traffic flow as the predicted value to be studied, use the existing data, combine the historical and future data relationship, predict the incomplete data, and then repair the missing data.

The classical k-nearest Neighbor (KNN) method fixes data by calculating the weighted average of the K Nearest neighbors of the missing value. KNN method is simple, easy to understand and implement, but its repair performance is highly dependent on the quality of the data around the missing data. Compared with KNN, Support Vector Regression (SVR) has a stronger generalization ability. SVR interpolates by finding a hyperplane that minimizes the distance from the observed value to the plane. This method assumes highly similar traffic flows for several consecutive days. Therefore, it cannot be adjusted to random changes in daily traffic flow.

Compared with traditional machine learning, deep learning models can automatically learn feature representations from data, better adapt to large-scale and high-dimensional data, and have strong generalization ability, which has gradually become an important direction of research and application. Tian *et al*. [25] proposed a method based on short and long term memory network for time series data completion. Cao *et al*. [68] proposed a driven interpolator Brits that could handle multiple correlated missing values and nonlinear time series. Wang *et al*. [72] analyzed the spatio-temporal correlation characteristics of traffic speed and proposed a data repair method based on Gated Recurrent Unit (GRU). Kong *et al*. [73] proposed a data repair method based on Dynamic Graph Convolutional Gated Recursive Unit (DGCGRU) by considering the dynamic spatial characteristics of traffic flow in road network. Zhao *et al*. [74] propose a time-graph Convolutional Network based on Graph Convolutional Network (GCN) and GRU, which extracts spatio-temporal features through the convolution layer of spatial graph and the two-dimensional convolution layer of time. Dong *et al*. [75] proposed a repair method based on tensor combination time similarity revisiting graph convolutional gated recursive unit (T-TRGCGR). Lu *et al*. [76] proposed a repair method based on improved Tucker decomposition, which can effectively consider the spatiotemporal correlation of traffic flow between lanes and has a good model repair accuracy. Generative Adversarial Network (GAN) [77], as a representative of generative models in deep learning, is widely used in the field of data repair. Yoon *et al*. [80] proposed Generative Adversarial Imputation Networ (GAIN) to generalize Gans, which use a prompt matrix based on real observations for unsupervised data generation and can run successfully even without complete data.

Since Long short-term memory (LSTM) has good temporal memory ability and can extract the internal relationship between serial data, it is often used in the research of traffic flow data repair. A large number of studies have proved the rationality of applying LSTM to data repair based on temporal and spatial characteristics of traffic flow. For example, Zhao *et al*. [69] firstly used deep learning to extract rules from historical data and perform data interpolation, and then used LSTM model to predict data and verify the validity of data interpolation. Hu *et al*. [19] used multi-layer LSTM module to extract daily and weekly spatio-temporal cycle features, and considered the time characteristics of traffic flow. Yan *et al*. [70] analyzed the spatial correlation of traffic flow data by using skidp-Gram model, and proposed a BILSTM-based repair method. Zhao *et al*. [71] combines graph convolution with Bi-LSTM to capture spatiotemporal dependencies, but applying GCN to extract spatial features may not be suitable for simple road sections.

The prediction-based missing data repair can effectively extract and capture the spatiotemporal change rules and relevant features of traffic flow, but it fails to consider the correlation of attributes among the influencing factors of traffic flow, and ignores the difference between the incomplete part of the data and the predicted data value, resulting in a large difference between the predicted data value and the missing real data value. Moreover, the higher the proportion of missing data, the greater the disparity.

At present, the application of deep learning models to repair traffic flow data has great advantages, and can effectively extract and capture the spatiotemporal change rules and relevant features of traffic flow. However, it also faces some challenges. For example, most models do not consider the correlation of attributes among factors affecting traffic flow, and ignore the difference between the incomplete part of data and the predicted data value. There is a large difference between the predicted data value and the missing real data value, and the higher the proportion of missing data, the greater the difference.

Scholars have proposed a lot of data repair methods and achieved a lot of excellent results, but there are still some shortcomings:

(1) From the perspective of time, most studies repair traffic flow data based on the traffic flow data under a long sampling period (as shown in Table 1). It is difficult to effectively identify the wrong data with small errors and not obvious enough, and it is difficult to properly consider the long-term and short-term dependence and time correlation between data, which affects the repair effect of missing data.
(2) From the perspective of space, due to the fact that detectors are not densely arranged in the actual traffic environment, it is often difficult to make good use of the spatial correlation between traffic flow data. Especially when the distance between adjacent sampling points is relatively long, the repair effect of missing data based on spatial similarity is often poor, which will affect the effect of data repair and increase the difficulty of data quality control.
(3) From the perspective of method, deep learning method has better application effect, and traditional interpolation method also has certain advantages, such as more intuitive method, but there are few methods to repair traffic flow data by combining the two methods; At the same time, different missing data types (such as random missing and continuous missing) have different characteristics, but most studies use a single repair strategy. The above two points make the repair method not strong robustness, repair effect is not ideal.

Based on this, a traffic flow data quality control method at video frame rate considering the segmental geospatial similarity is proposed. Based on the traffic flow data at video frame rate, this method uses the extremely high spatial similarity of cross-sectional traffic flow data to repair the traffic flow data, aiming at the different characteristics of random and continuous missing data. At the same time, interpolation method and deep learning method are used to repair traffic flow data to improve the repair effect of traffic flow data.

## Methodology

In order to improve the spatio-temporal availability of traffic flow data, traffic flow data is collected based on traffic flow surveillance video to improve the temporal resolution and spatial density of traffic flow data firstly. Secondly, the quality analysis of traffic flow data is carried out to effectively identify the data that need to be repaired. Further, based on the cross-sectional geospatial similarity, the continuous missing data is preprocessed, and the piecewide interpolation repair of random missing data and the multi-sectional combined repair based on LSTM are carried out to effectively control the traffic flow data.

### Video-based multi-section traffic flow data collection

#### Video-based traffic flow collection parameter setting.

1. Sampling frequency

Based on the high update rate of video images, the sampling time of vehicle information can be accurate to the frame level, so the sampling frequency of traffic flow data can also be accurate to the video frame.

2. Sampling point

**Fig 1:**
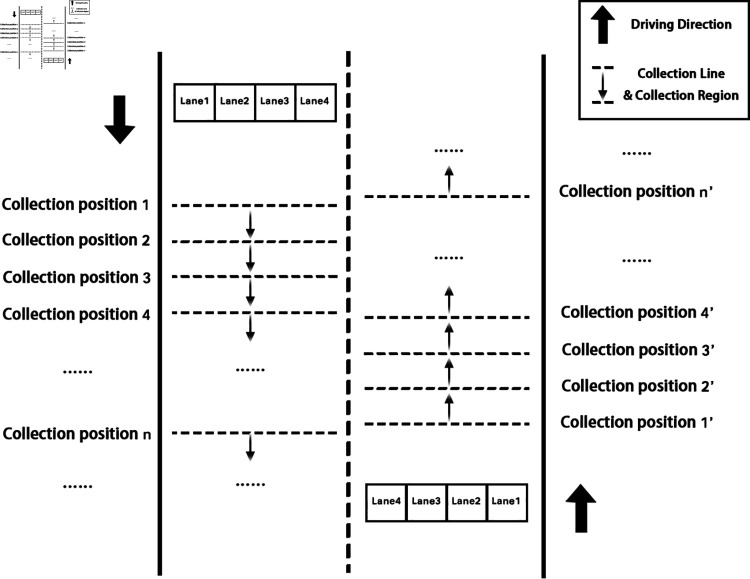
Schematic diagram of spatial information coding.

In order to improve the spatial density of traffic flow data, sampling points will be arranged on several road cross-sections within the video image range, and the serial number of each sampling point will be encoded along the vehicle direction. Fig 1 shows the schematic diagram of spatial information coding. As shown in the figure, the sampling line is a detection line parallel to the lane at each sampling point, and the sampling region is the area between two adjacent sampling lines. Therefore, for the same lane, the number of sampling regions is one less than the number of sampling lines.

Taking a traffic surveillance video as an example, the multi-section layout of sampling points is carried out. Seven sampling points are arranged at fixed geographical intervals, and based on each sampling point, a sampling line is arranged for each lane along the direction perpendicular to the lane.

**Fig 2:**
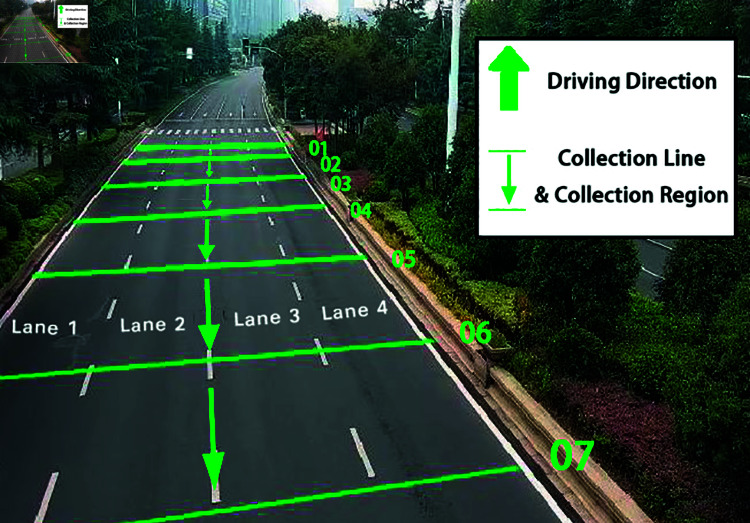
Layout of sampling points for traffic flow data.

Fig 2 shows the layout of sampling points of the video base station. It should be noted that since the basic principle of camera imaging is pinhole imaging, roads in traffic surveillance videos usually have certain deformation, so that sampling lines with fixed geographical intervals are mapped to the image in a state of dense in the distance and sparse in the near, and are not parallel. The specific conversion method of image pixel coordinates and geographic coordinates will be elaborated in the next section.

#### Transformation of image pixel coordinates and geographic coordinates.

Next, the image pixel coordinates and geographical coordinates are converted. In this paper, the road marking is taken as reference, and the polynomial interpolation method is used to carry out two-dimensional interpolation, which realizes the correspondence and transformation of image pixel coordinates and geographical coordinates. The mapping formula for coordinate transformation is as follows:

T:A→B (1)

Where, *A* is the set of coordinates of image pixels, namely (x,y)∈A; *B* is the set of geographical coordinates, namely $(x^{\prime },y^{\prime })\in B$; *T* is the corresponding law of image pixel coordinates and geographical coordinates. Further, the relationship between (x,y) and $\left(x^{\prime },y^{\prime }\right)$ can be expressed as:

$$\left(x^{\prime },y^{\prime })=T(x,y\right)$$ (2)

#### Vehicle detection.

In order to improve the efficiency of vehicle detection, a detection region is set according to the collection region of traffic flow information within the image range, that is, only the vehicles in this region are identified and extracted.

**Fig 3:**
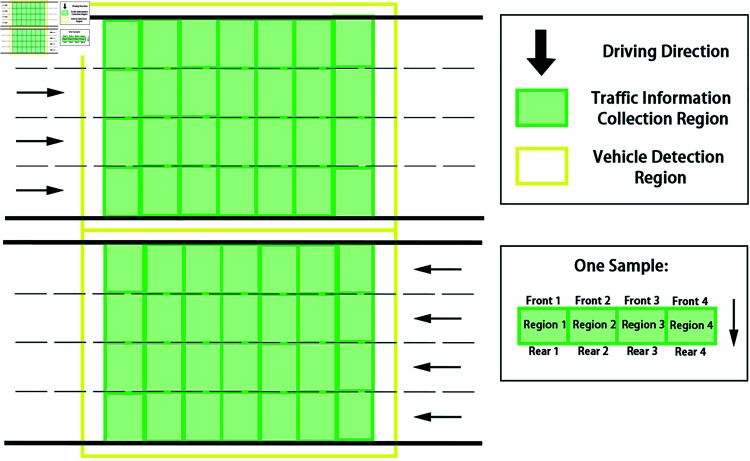
The description of traffic flow data collection and vehicle detection region.

Take a four-lane road cross-section as an example. Fig 3 shows the description of traffic flow data collection and vehicle detection region. As shown in the figure, the black arrow is the driving direction; The green region is the lane collection region of traffic flow information, and *Region*_*i*_ is the collection regions on each lane, respectively. *Front*_*i*_, *Rear*_*i*_ are the detection lines for vehicle arriving and leaving the collection region on each lane, respectively. The yellow region is the vehicle detection region, which is slightly larger than the green region.

**Fig 4:**
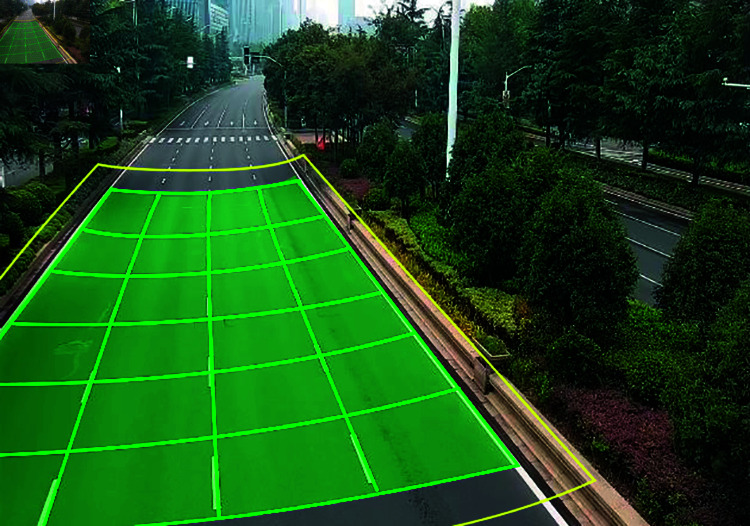
Layout of the detection region.

As the basic principle of camera imaging is small hole imaging, the road in traffic surveillance video usually produces a certain deformation, so the traffic flow information collection region and vehicle detection region will show a trapezoidal shape, as shown in in Fig 4.

**Fig 5:**
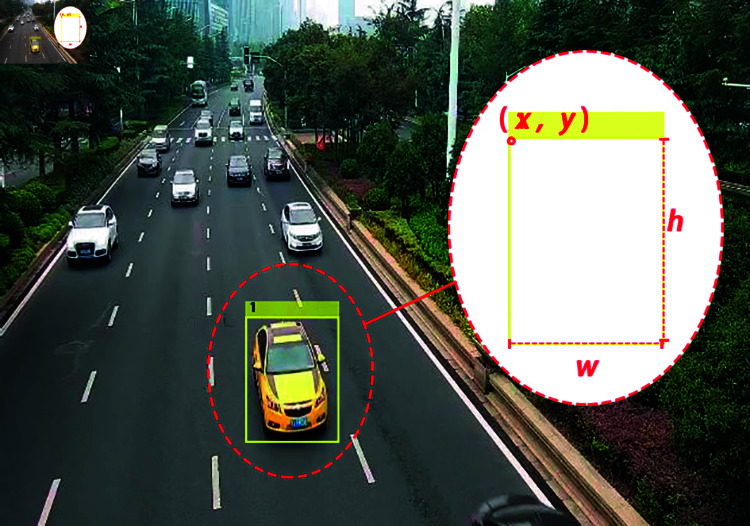
Storage of vehicle information.

In this paper, deep neural network YOLOv4 is used as vehicle detection model. The transfer learning is carried out based on YOLOv4 with pre-trained ResNet50 as the basic model. For the storage of vehicle information, the size and location of the vehicle detection box are recorded. As shown in Fig 5, the information of a vehicle detection frame contains four elements: the horizontal coordinate (*x*) and vertical coordinate (*y*) in the upper left corner of the vehicle detection frame, and the (*w*) and height (*h*) of the vehicle detection frame, which are stored in vector form. The expression is as follows:

bbox=[x,y,w,h] (3)

#### Traffic flow information collection.

When the vehicle reaches the front detection line *Front*_*i*_ on any lane (*i* is the lane number), the detection region *Region*_*i*_ is activated, and the moment of triggering the front detection line *Front*_*i*_ is recorded. When the vehicle arrives at the rear detection line *Rear*_*i*_, the moment when the vehicle triggers the rear detection line *Rear*_*i*_ is recorded, and the traffic flow information of the vehicle is considered to be collected and stored as a vehicle information. At the same time, if the vehicle enters the region during the process of activating the detection region *Region*_*i*_, the vehicle information is stored in the order of the detection lines before triggering.

Collect traffic flow data at the sampling frequency of video frame rate:

(1) Flow

Flow is the number of vehicles observed in the time granularity converted to the number of vehicles in one hour, calculated as follows:

$$Q_{i}(t)=\frac{N_{i}}{T}\times 60$$ (4)

Where *Q*_*i*_(*t*) refers to the traffic flow on lane *i* at time *t*; *T* is the time granularity, and the time granularity of 15 min is selected for aggregation of traffic flow data; *N*_*i*_ represents the number of vehicles passing on lane *i*.

(2) Speed

Speed is a comprehensive measure of the speed of all vehicles in the traffic system under study, with the average speed of the interval as the traffic speed, and the calculation formula is as follows:

$$V_{i}(t)=\frac{1}{\frac{1}{N_{i}}\sum_{j=1}^{N_{i}}\frac{1}{v_{j}}}$$ (5)

Where, $V_{i}(t)$ refers to the traffic speed on lane *i* at time *t*; *N*_*i*_ represents the number of vehicles passing through the detection region *Region*_*i*_; $v_{j}$ refers to the speed of the *j*^*th*^ vehicle, which is calculated as follows:

$$v_{j}=\frac{L_{i}}{t^{R}-t^{F}}$$ (6)

Where, *L*_*i*_ is the length of collection region *Region*_*i*_; *t*^*F*^ and *t*^*R*^ are the moments when the vehicle triggers the pre-detection line *Front*_*i*_ and the post-detection line *Rear*_*i*_, respectively.

(3) Density

Density is the harmonic average of traffic density in the time granularity, calculated as follows:

$$K_{i}(t)=\frac{1}{\frac{1}{N_{i}}\sum_{j=1}^{N_{i}}\frac{1}{k_{j}}}$$ (7)

Where, *K*_*i*_(*t*) refers to the traffic density on lane *i* at time *t*; *N*_*i*_ represents the number of vehicles passing through the detection region *Region*_*i*_ in the time period [T−t×60,t]; *k*_*j*_ refers to the *j*^*th*^ density and is calculated as follows:

$$k_{j}=\frac{n_{j}}{L_{i}}$$ (8)

Where, *L*_*i*_ is the length of collection region *Region*_*i*_; *n*_*j*_ is the number of vehicles in the *Region*_*i*_ at time *t*^*F*^.

### Quality analysis of traffic flow data

The premise of traffic flow prediction is to collect accurate traffic flow data, and urban traffic flow data is affected by various factors such as people, vehicles, roads and environment, and therefore presents certain complexity. Due to technical factors such as vehicle detection errors, or hardware factors such as video failure, the raw traffic flow data collected will have various quality problems. In order to analyze the traffic flow data more accurately, it is necessary to analyze and control the quality of the collected traffic flow data in view of the possible quality problems.

#### Missing data.

The video-based traffic flow data collection technology proposed in this paper collects traffic flow data according to a certain time interval. The black screen caused by video failure, line transmission and other reasons makes the vehicle detection technology based on video unable to detect vehicle data normally, resulting in traffic flow data at a certain time or period of data missing.

**Fig 6:**
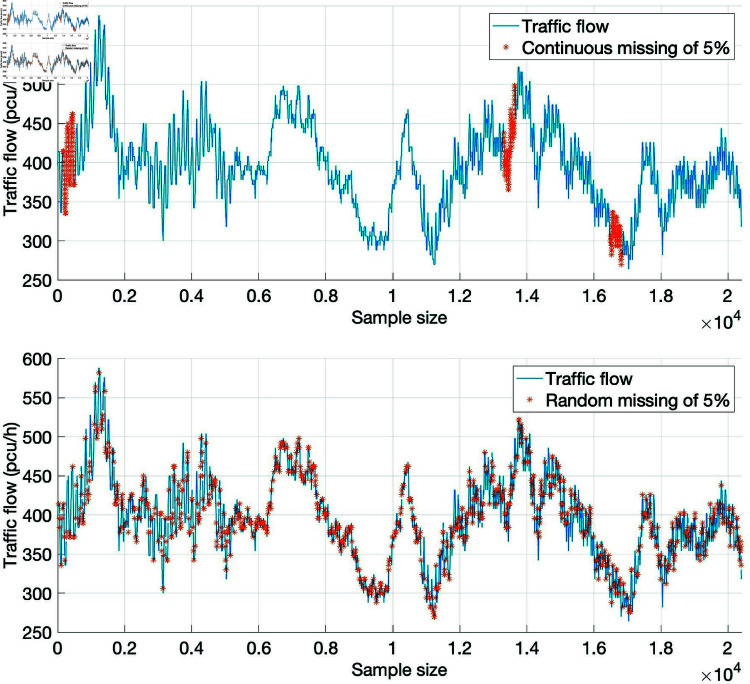
Example of missing data.

According to the frequency and duration of video failure, missing data can be divided into two kinds of missing data: random missing and continuous missing. Random missing means that the location of missing data is more scattered, which is more common and less difficult to repair. For continuous missing data, because the missing location is more concentrated, it is difficult to repair. As shown in Fig 6, under the same data missing rate (5%), random missing is easier to repair the missing data by combining the relationship between the data because the missing data locations are scattered. For continuously missing data, the difficulty of repair is increased because the missing location is more concentrated.

#### Wrong data.

The collected data may be wrong due to errors in vehicle detection or faults in transmission network. The wrong data usually does not conform to the overall change trend of the data, and the difference between the data and the neighboring data is large, which does not conform to the reasonable range of traffic flow data.

When a large amount of wrong data is collected, simply eliminating all wrong data may lead to the missing of traffic flow information, and thus cannot accurately describe the characteristics of traffic flow. Therefore, it is necessary to first identify the wrong data from the collected data, and then repair the wrong data.

1. Preliminary screening based on road design principles

Each city has graded the roads, and specified the corresponding design speed and basic road capacity. In this paper, design speed and basic road capacity are taken as the threshold conditions of flow and speed respectively. When the collected flow and speed exceed the design speed and the basic traffic capacity of the road within a certain range, the flow or speed at that moment is set as a null value and is regarded as missing data. Set the thresholds for flow (q), speed (v), and density (k) as follows:

$$0\leq q\leq f_{q}\cdot C_{B}$$ (9)

$$0\leq v\leq f_{v}\cdot S_{D}$$ (10)

k≥0 (11)

Where, *C*_*B*_ is the basic traffic capacity of the road (pcu/h); *S*_*D*_ is the design speed of the road (km/h); *f*_*q*_ is the correction coefficient of flow rate, which is 1 - 1.2; $f_{v}$ is the correction coefficient of velocity, which is 1.2 - 1.5.

2. Comprehensive judgment based on traffic flow theory

Threshold filtering can initially eliminate abnormal data that is not within the reasonable range, but it can not completely solve the problem of identifying wrong data. For example, when a parameter is 0, the threshold value of a single traffic flow feature parameter cannot accurately determine whether the data is reasonable, so it is necessary to make a comprehensive judgment by combining three traffic flow feature parameters. Secondly, the threshold range of density is too broad to accurately judge the rationality of density.

On this basis, a comprehensive judgment will be made according to the traffic flow theory and three traffic flow characteristic parameters. Firstly, the parameter relationship between flow rate, velocity and density is used to narrow the range of density threshold. Secondly, according to the actual driving state of the vehicle on the road, the evaluation standard of logical discrimination is established.

(1) Threshold setting of density

Under ideal conditions, there is a certain basic relationship between the characteristic parameters of traffic flow (flow, speed and density), that is

$$Q=\bar{V_{s}}\cdot \bar{K}$$ (12)

Where: Q is the average flow (pcu/h); $\bar{V_{s}}$ is the interval average speed (km/h); $\bar{K}$ is the average density (veh/km). Based on this relationship, the threshold range of density(k) reduction is further determined:

$$f_{k}^{min}\cdot \frac{q}{v}\leq k\leq f_{k}^{max}\cdot \frac{q}{v},\;\;\;q,u>0$$ (13)

Where, q and v are the flow and speed collected at the current time respectively; $f_{k}^{min}$ and $f_{k}^{max}$ are the density correction coefficients, which are 0.5 and 1.5 respectively.

When the traffic flow is blocked, the flow or speed will be the value of "0", the threshold condition fails, at this time, the density should be higher than the optimal density when the flow and speed reach the peak, which can be expressed as a formula:

$$k\geq f_{k}\cdot \frac{C_{B}}{S_{D}}$$ (14)

Where, *C*_*B*_ is the basic traffic capacity of the road (pcu/h); *S*_*D*_ is the design speed of the road (km/h); *f*_*k*_ is the density correction coefficient, which is 0.8.

(2) Evaluation criteria of logical discrimination

When there is no vehicle passing through the road, the three traffic flow characteristic parameters are all "0" values. When the road is open for traffic, the three traffic flow characteristic parameters are all positive. When the road is blocked to the point that the vehicle cannot move, the flow and speed are "0" values, and the density should be larger. When the traffic flow data at a certain moment does not meet any of the above three combinations of traffic flow characteristic parameters, the traffic flow data at that moment can be judged as abnormal data and set to null.

### Design of traffic flow data quality control method

The above introduces the traffic flow data quality control analysis method to realize the identification of missing data and wrong data, so as to carry out targeted data repair. Considering that different data repair methods have different characteristics, advantages and disadvantages, a combined repair model considering cross-sectional geospatial similarity is designed.

**Fig 7:**
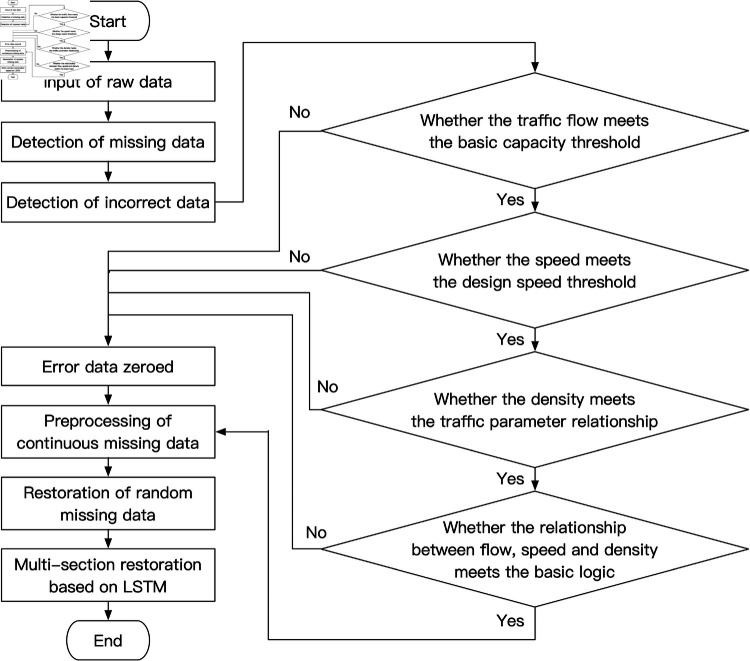
Flow chart of traffic flow data quality control method.

Firstly, based on the data similarity of traffic flow data at multi-cross sampling points, the continuous missing data is transformed into random missing data or short continuous missing data. Secondly, for different missing points, the piecewise low-order interpolation polynomial with the lowest error is selected as the piecewise low-order interpolation polynomial to repair the missing points. Finally, taking the current multi-section traffic flow data as a whole, LSTM model is used to predict the complete data at the current moment. Fig 7 shows the flow of traffic flow data quality control method:

#### Cross-sectional geospatial similarity analysis.

Section similarity refers to the similarity analysis between traffic flow time series on different cross-sections of the same road cross-section. Pearson correlation coefficient was used to calculate the cross-correlation between traffic flows at different time granularity. Traffic flow data corresponding to the same time on different cross-sections are extracted for calculation. The calculation formula is as follows:

$${\hat{R}}_{x^{S_{i}}x^{S_{j}}}=\frac{\sum_{t=1}^{T}x_{t}^{S_{i}}x^{S_{j}}}{\sqrt{\sum_{t=1}^{T}x_{t}^{S_{i}}x^{S_{i}˙}\sum_{t=1}^{T}x_{t}^{S_{j}}x^{S_{j}}}}$$ (15)

Where, T is the length of the time series and t is the serial number of the time series. *S*_*i*_ and *S*_*j*_ represent different cross-sections. $x^{S_{i}}$ and $x^{S_{j}}$ are the traffic flow data on different cross-sections, respectively.

Considering that in this paper, traffic flow data on the road is collected and data quality control is carried out based on road surveillance video, the road length within the scope of a single video image is limited, generally within 300 meters. In this study, the interval of the collected cross-sections was set to about 15 meters to ensure that the adjacent cross-sections had high similarity.

**Fig 8:**
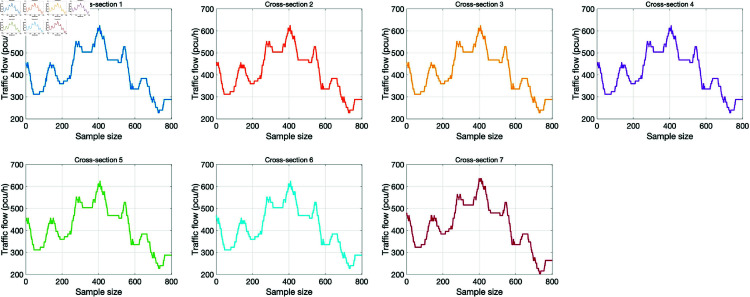
Traffic flow data on different cross-sections.

Fig 8 respectively shows the traffic collected at different cross-sections in the same time period. It can be found that the traffic flow data changes at each collection point are very similar. Due to the spatial sequence of cross-sections, the traffic flow data collected from different cross-sections at the same time have a certain lag relation in time. The traffic flow data is translated appropriately, and then the similarity of traffic flow data between cross-sections under different horizontal transition lengths is compared. The highest similarity of horizontal transition lengths is the lag relation of traffic flow data between cross-sections.

**Fig 9:**
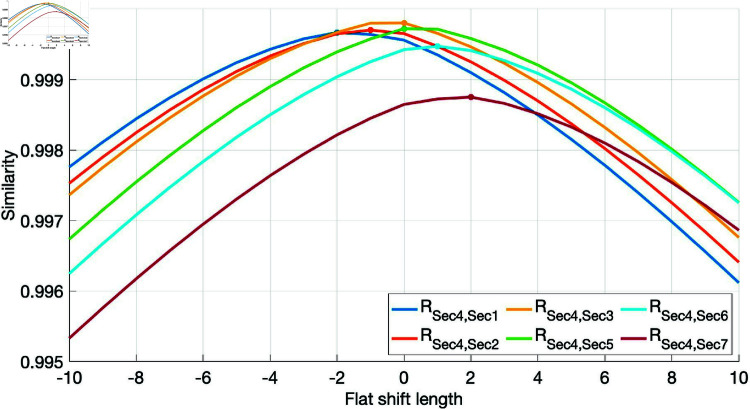
Similarity of traffic flow data between cross-sections with different horizontal transition lengths (cross-section 4).

Fig 9 shows the similarity of traffic flow data between cross-section 4 and the other six cross-sections at flat step length [-10,10]. The maximum similarity is marked with "*". Overall, the data of cross-section 4 and cross-section 3 are the most similar, followed by cross-section 5, then cross-section 1 and 2, and finally cross-section 6 and cross-section 7. The lag relation is 0 step, 0 step, -1 step, -2 step, 1 step and 2 step respectively. Based on this, it can be found that the closer the two cross-sections are in space, the fewer the lag steps and the higher the data similarity, which is in line with the expectation. Based on the similarity of traffic flow data between cross-sections, the missing data will be repaired.

#### Pretreatment of continuous missing.

For continuous missing data, the key to repair is to select appropriate data to fill in the continuous missing interval, interrupt the continuous missing interval to narrow the scope of the continuous missing interval, and transform the continuous missing into random missing or shorter continuous missing.

When the traffic flow data on a cross-section is missing in a certain continuous period, the data similarity between this cross-section and other cross-sections is analyzed in a slightly larger continuous missing period. Select the cross-section with the highest similarity and high data integrity, fill in some missing data, and transform continuous missing into random missing or short continuous missing. Then, the processing method of repairing random missing data is used to repair the remaining missing data.

**Fig 10:**
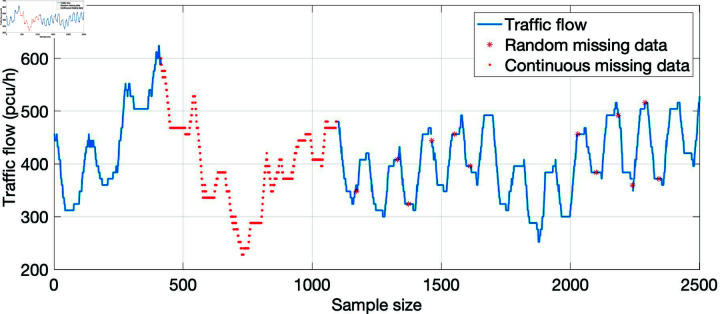
Traffic flow data on cross-section 3.

**Fig 11:**
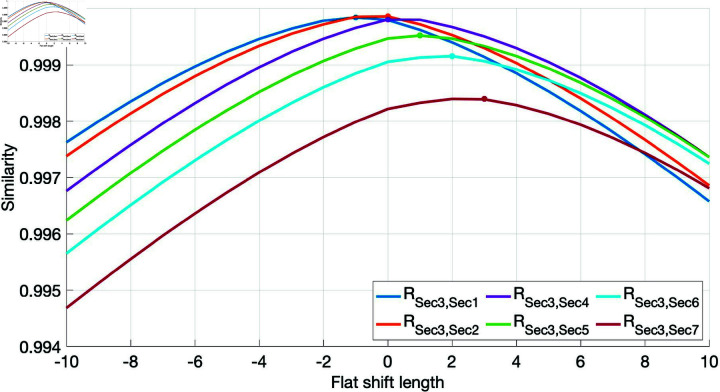
Similarity of traffic flow data between cross-sections with different horizontal transition lengths (cross-section 3).

Fig 10 shows a cross-section of cross-section 3 with continuous missing data. The data similarity between cross-section 3 and the rest of cross-sections is analyzed in a period slightly longer than the continuous missing period, and the analysis results of data similarity with different horizontal step lengths are shown in Fig 11. As shown in Fig 11, the data of cross-section 3 and cross-section 2 are the most similar and the shift step length is 0 steps.

**Fig 12:**
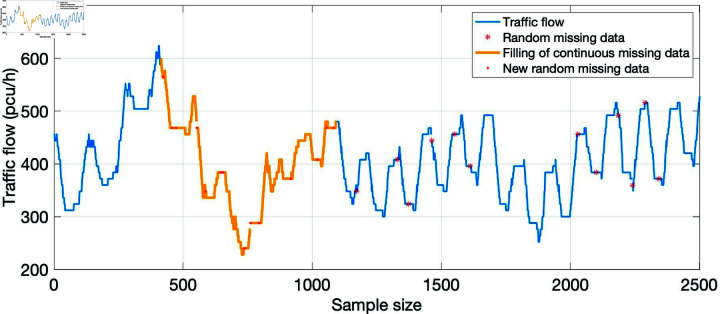
Filling of continuous missing data on cross-section 3.

Based on the data of cross-section 2, the continuous missing interval of cross-section 3 can be partially filled, which can transform the continuous missing into random missing or short continuous missing, and improve the repair effect to a certain extent. The filling results are shown in Fig 12.

According to the experimental research in this paper, the similarity of adjacent cross-sections on the same road cross-section is above 0.95. Considering that the similarity of traffic flow data between cross-sections will decrease in the case of missing data, the similarity threshold is set at 0.8 in this paper. When the similarity is lower than the threshold, skip this step and directly enter the repair step of random missing.

#### Piecewise interpolation repair based on cross-sectional geospatial similarity.

When the traffic flow data on a cross-section is missing at a certain time, the data similarity between this cross-section and other cross-sections is analyzed on the interpolation interval of the missing point. Select the cross-section with the highest similarity and complete missing point data, and use different segmented low-order interpolation polynomials to fit the traffic flow data on the adjacent cross-section. By comparing the fitting errors under different piecewise low-order interpolation polynomials, the piecewise low-order interpolation polynomial with the lowest error is selected as the piecewise low-order interpolation polynomial to repair the missing point.

#### Multi-section combined repair model based on LSTM.

In view of the strong spatial correlation between the multi-section traffic flow data, the multi-section traffic flow data is considered as a whole, and the LSTM data repair model is constructed. The basic idea is to fully combine LSTM’s good time memory ability and the ability to extract the internal relationship of sequence data, and predict the complete data at the current moment based on the preliminary repair results of "Pretreatment of continuous missing" and "Piecewise interpolation repair based on cross-sectional geospatial similarity", so as to achieve more accurate data repair.

1. Data preparation

In order to make the repair model more robust, it is proposed to use different missing rates for random missing processing of complete traffic flow data, including 5%, 10%, 15%, 20%, 25%, 30% and so on.

For random missing, we select a set of random seeds with the same number of missing values within the length range of the time series, and take the position corresponding to the random seed value as the position of the random missing value. For continuous missing, we generally construct continuous missing at two positions in a set of time series. The specific method is to select two groups of random seeds, and each group of random seeds is two. Among them, the first group of random seeds is used to represent the location of continuous missing, and the second group of random seeds represents the length of continuous missing. Cause the computer to continuously produce two random seeds whose sum equals the number of missing values.

Through experimental comparison, it is found that there is little gap between the missing data processed manually and the real data collected in the actual scene, and the missing data obtained is more difficult to repair.

2. Network structure and parameter setting

Compared with other deep learning models, recurrent neural networks (RNN) have great advantages in processing time series. In order to solve the problem of gradient disappearance or gradient explosion when RNN process long sequences, the researchers proposed LSTM. Through the introduction of gating mechanism and memory unit, LSTM can better deal with long sequence data and learn long-term dependencies, thus improving prediction accuracy, and showing excellent performance in processing sequence data. Based on the above reasons and considering the nonlinear and stochastic characteristics of traffic flow time series, LSTM is used as the baseline model for optimizing data repair.

**Fig 13:**
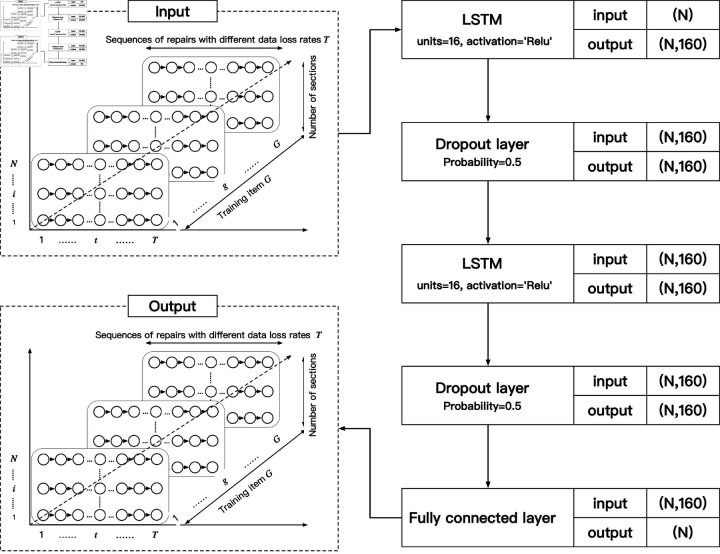
Structure and parameter settings of the repair model.

In order to better train the data repair model, a 2-layer LSTM model is designed for training. The structure and parameter settings of the model are shown in Fig 13.

(1) Input Layer

The input layer is used to input the training data of the model. The quantity, structure and quality of the samples used for training all have a crucial impact on the structure of the model and the training effect.

- Length of time series

Traffic flow is a time series, so the most important thing for the characterization of traffic flow time series is the length of time series suitable for training, which should not be too short or too long. When the time series is too short, its changing trend is difficult to describe the traffic flow state of a period of time, and it is difficult to meet the requirements of the deep learning network on the number of samples. When the time series is too long, it contains too many historical features, which is not suitable for short-term traffic flow prediction, but also produces a large amount of computation and a long training time, and even leads to the appearance of "overfitting" which affects the training effect of the model. The study intends to select the appropriate time series length within 20 minutes to 40 minutes.

2. Data processing and data mode

Firstly, the traffic flow data on multiple cross-sections are selected, and the complete original data on multiple cross-sections are processed by random missing with different missing rates, and continuous and random missing data are constructed respectively. Secondly, the continuous missing data is preprocessed to transform the continuous missing into random missing or shorter continuous missing. Thirdly, the piecewise low-order interpolation polynomial with the lowest selection error is used as the piecewise low-order interpolation polynomial to repair the missing data. Finally, a multi-section input matrix with spatial attributes is constructed to train the LSTM model.

Fig 13 shows the input data mode of the model in tensor form. The horizontal representation describes variably long time series, with each time point represented by *t*, and variably long time series represented by *T*. The ordinate representation describes the time series features of traffic flow in multi-section. Each time series feature is described by *i*, and the number of cross-sections is represented by *N*. The third dimension is training programs. Each set of training items is represented by *g*, and a total of 500 training items of different time series lengths are represented by *G*.

The input of a single variable is represented by *X*, and each element in *X* is represented by *x*_*t*,*i*,*g*_, which means the specific value of the timing feature *i* at the time point *t* in the training item *g*.

(2) LSTM layer

In this study, 160 hidden units are set up in the LSTM layer, the input size of the LSTM layer is the number of features *N* and the output size 160.

The other hyperparameters that need to be determined include learning rate, etc., which will be adjusted based on Bayesian Optimization algorithm (BO). BO is a hyperparameter optimization algorithm with good performance. Different from grid search and random search, Bayesian optimization uses prior information and observation results to define the posterior distribution of function space, and can describe some characteristics of the objective function through prior information under the condition of uncertain form of the objective function, so that Bayesian optimization can optimize hyperparameter values more efficiently with fewer iteration times and faster speed.

In order to better select the combination of hyperparameters, a hyperparameter search space to optimize the selection of hyperparameters is set up in a certain range. The optimized hyperparameters include network depth, number of hidden layer nodes, initial learning rate, random gradient descent momentum, L2 regularization intensity, and other hyperparameters are set according to parameter tuning experience. Table 2 lists the specific settings. We set the same hyperparameters for both layers of LSTM, and select Mean Square Error (MSE) as the loss function.

**Table 2:** Ranges of hyperparameters.

Name	Parameter value or range	Parameter results
Network depth	[1,10]	5
Initial learning rate	[10^−3^,10^−1^]	0.03846
Random gradient descent momentum	[0.80,0.98]	0.85923
L2 regularization intensity	[10^−10^,10^−2^]	1.3924e-06
Training Max rounds		60
Small batch size		256

Further, the weight matrix and activation function in the LSTM model are shown in Table 3.

**Table 3:** The weight matrix and activation function in the LSTM model.

Layer name	Weight matrix		Bias	Activation function	
-35-6	InputWeights	RecurrentWeights		State	Gate
lstm_1	640 × 6	640 × 160	640 × 1	tanh	sigmoid
lstm_2	640 × 160	640 × 160	640 × 1	tanh	sigmoid

(3) Dropout Layer

Setting a dropout layer in the model changes the underlying network architecture between iterations, helping to prevent network overfitting. The key idea of dropout technology is to randomly remove units (and their connections) from the neural network during training, and the greater the probability of dropping, the more elements are dropped during training.

In general, each cell is retained with a fixed probability pindependent of the others, where *p* can simply be set to 0.5, which is a reasonable approximation to taking the geometric mean of the predictive distributions produced by the exponentially-many drop out networks [78], which is also close to the optimal value for a wide range of networks and tasks [79]. With that in mind,the probability of the dropout layer is set to 0.5.

(4) Fully Connected Layer

The dropout layer does not change the input and output between layers, so the input size of the fully connected layer is as same as the output size of the LSTM layer, and the output size of the fully connected layer depends on the output layer.

(5) Output layer

The output layer is used to output the predicted data of the model, and the data mode is similar to the input, except that the element is the predicted data. The output of a single variable is represented by *Y*, and each element in *Y* is represented by *y*_*t*,*j*,*g*_, which means the traffic flow prediction data at the time of (*t*) in the training item *g*.

The difference with the traffic flow prediction is that the output here is not the prediction of the future moment, but the prediction of the current moment to output the repair result of the traffic flow data, that is, the complete original traffic flow data of multiple cross-sections at the current moment is used as the prediction target.

## Experimental results and discussion

### Experimental data and evaluation indexes

#### Experimental data.

The video data of this paper comes from the traffic video base stations set on the road. The selection period of video data is from October 2021 to October 2022. In this period, 120 pieces of continuous video data with a duration of 6 hours are randomly selected, that is, 720 hours of video data. The 120 video data selected at random included the video data of 12 traffic video base stations, and each traffic video base station collected 10 video data for a total of 60 hours.

1,200 groups of traffic flow data with a time series length of 20 minutes to 40 minutes are randomly selected from the above 120 videos, and each video contains 10 groups of experimental data. The surveillance videos in this paper are all located in the main urban area of Nanjing. Compared with expressways and urban fast roads, the data missing is more frequent and the randomness of traffic flow sequence is greater, so it is more difficult to repair.

#### Evaluation index.

RMSE is the square root of the ratio of the square of the difference between the observed value and the true value and the number of observed values *n*. RMSE is calculated as follows:

$$RMSE=\sqrt{\frac{1}{n}\sum_{t=1}^{n}{\left(\hat{y}(t)-y(t)\right)}^{2}}$$ (16)

Where, *RMSE* is the root mean square error, *n* is the number of observed values, $\hat{y}(t)$ is the observed value of time node *t*, and y(t) is the real value of time node *t*.

### Analysis of data quality control effect

In order to verify the effectiveness of the algorithm, we use the correct traffic flow data collected as the validation set, and compare the accuracy of traffic flow data before and after data quality control.

**Fig 14:**
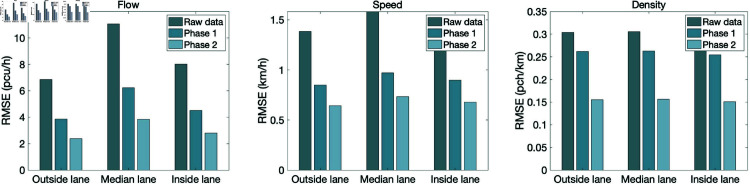
Analysis of quality control effect of traffic flow data.

Fig 14 shows the quality control effect analysis of traffic flow data on different lanes. In the figure, "Phase 1" refers to piecewise interpolation repair based on cross-sectional geospatial similarity, and "Phase 2" refers to multi-sectional combined repair based on LSTM. In order to more intuitively and reasonably compare the accuracy before and after data quality control, RMSE, which has the same dimension as the original data and is more sensitive to errors, is used as the evaluation index in this section.

On the whole, with the deepening of data quality control, the accuracy of traffic flow data on each lane has been improved to a certain extent, and the gap between the accuracy of traffic flow data between different lanes has become smaller. At the same time, compared with speed and density, the improvement of flow accuracy is more obvious.

### Comparative experiment

At the same time, in order to better illustrate the feasibility of the proposed data quality control method and analyze the influence of different sampling periods on data repair, Lagrange interpolation and four piecewise low-order interpolation methods (nearest neighbor interpolation, piecewise linear interpolation, piecewise parabola interpolation, cubic spline interpolation) are selected as the comparison model of the proposed algorithm.

(1) Lagrange interpolation

Lagrange interpolation is a polynomial interpolation method named after Joseph Lagrange, a French mathematician in the 18th century. The method can find a polynomial from the known observations so that it passes exactly through these observation points.

Any given 2*n* + 2 number *x*_1_, *x*_2_, ..., *x*_*n* + 1_, *y*_1_, *y*_2_, ..., *y*_*n* + 1_, where *x*_1_, *x*_2_, ..., *x*_*n* + 1_ is not identical, then there is a unique polynomial *p*_*n*_(*x*) of degree *n* that satisfies

$$p_{n}(x_{i})=y_{i},\;\;i=1,2,...,n+1$$ (17)

then *p*_*n*_(*x*) can be expressed as:

$$p_{n}(x)=\sum_{i=1}^{n+1}y_{i}(\prod_{j\neq i}^{1\leq j\leq n}\frac{\left(x-x_{j}\right)}{\left(x_{i}-x_{j}\right)})$$ (18)

This is the Lagrange interpolation formula.

(2) Nearest neighbor interpolation

Nearest neighbor interpolation is the easiest method to calculate. In this algorithm, every output value is the nearest original data sample value of the point, this interpolation method is also called point shift gorithm.

(3) Piecewise linear interpolation

The basic principle of piecewise linear interpolation â€Œ is to connect every two adjacent nodes with a straight line to form a broken line, which is the piecewise linear interpolation function. When calculating the interpolation of a certain point, only two nodes around the point are used, so the amount of computation is independent of the number of nodes, while the Lagrange interpolation is dependent on the number of nodes

(4) Piecewise parabola interpolation

Parabolic interpolation method, also known as quadratic interpolation method, is a polynomial interpolation method that approximates the minimum point of the original sought function by fitting the minimum point of the conic curve successively.

Specific practices are as follows: Let the function values of *f*(*t*) at $t_{1}<t_{2}<t_{3}$ be *f*(*t*_1_), *f*(*t*_2_) and *f*(*t*_3_) in turn, and use parabola

$$\phi (t)=a_{0}+a_{1}t+a_{2}t^{2}$$ (19)

to fit *f*(*t*), so that the equations composed of

$$\phi (t_{i})=a_{0}+a_{1}t_{i}+a_{2}t_{i}^{2},\;\;i=1,2,3$$ (20)

are satisfied, and the derivative of ϕ(t) is made to equal zero. $t=-a_{1}/2a_{2}$ is obtained, *a*_1_ and *a*_2_ are obtained from the above equations, and the formula for calculating the approximate minimum point can be obtained by substituting them into the solution. In each three-point group, the function value of the middle point *t*_2_ is not greater than the function value of the two endpoints of the search interval $t_{1},t_{3}$, and the search interval is gradually reduced through successive iterations. The iteration is terminated when the distance between the minimum points of two successive iterations is less than some predetermined distance, or when the difference between the value of the approximation function and the value of the original seeking function is less than some allowable error.

(5) Cubic spline interpolation

Cubic Spline Interpolation is a method of interpolation in which a smooth curve is constructed from a series of interpolation points. Mathematically, it obtains a set of curve functions by solving a system of three-moment equations. A cubic spline interpolation function is defined on the interval [a,b], given *n* + 1 nodes and a set of corresponding function values. The function satisfies $S(x_{i})=f(x_{i})$ at every node and has continuous first and second derivatives throughout the interpolation interval. On each cell $\left[x_{i},x_{i+1}\right]$, the cubic spline interpolation function is a cubic polynomial.

In addition, two machine learning algorithms were selected as comparison models, including KNN and GAN.

Traffic flow data with different missing rates (5%-30%) and different missing types (random missing and continuous missing) were repaired respectively, and the data quality control effects of traffic flow data with different missing rates and different missing types under different sampling cycles and different repair modes were compared and analyzed.

Take traffic flow as an example. First, the traffic flow data with a time resolution of 1 frame were sampled in 1 sec, 2 sec, 3 sec, 4 sec, 6 sec, 7 sec, 8 sec, 9 sec, 10 sec, 20 sec, 30 sec, 40 sec, 50 sec, 60 sec, 70 sec, 80 sec and 90 sec, and then processed into a total of 19 kinds of time resolution traffic flow data. Secondly, the missing rate of 5%, 10%, 15%, 20%, 25%, 30% was used to process the random missing and continuous missing of the above data. Then, Lagrange interpolation, four piecewise low-order interpolation methods and multi-section combined repair method based on LSTM were used to repair the above traffic flow data. The RMSE value was also used as the evaluation standard.

**Fig 15:**
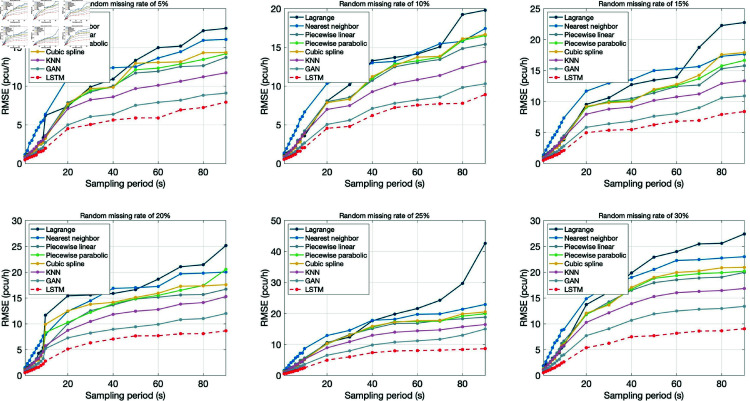
Comparison of random missing data repair errors.

**Fig 16:**
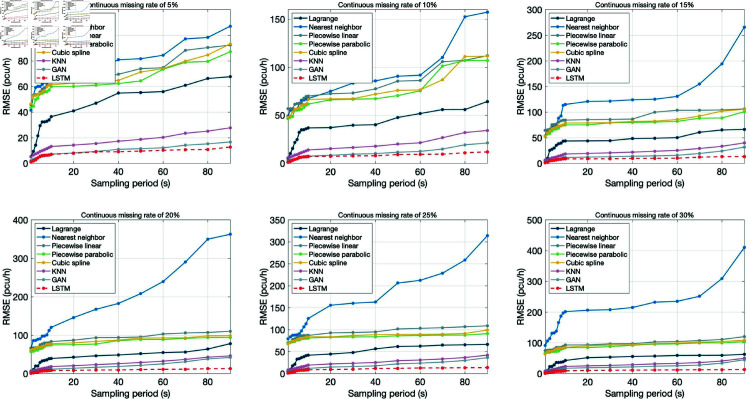
Comparison of continuous missing data repair errors.

Figs 15 and 16 respectively show the repair errors of traffic flow data with sampling period from 1 frame to 90 seconds by using different repair models under random missing and continuous missing. In the figure, the horizontal coordinate represents the sampling period, and the vertical coordinate represents the RMSE value of the data repair method. Each subgraph shows the data repair situation with data missing rate of 5%, 10%, 15%, 20%, 25% and 30% respectively.

In general, the multi-section combined repair method based on LSTM proposed in this paper has the best repair effect, followed by two machine learning algorithms GAN and KNN, then piecewise linear interpolation, piecewise parabola interpolation and cubic spline interpolation, and finally, nearest neighbor interpolation and Lagrange interpolation.

Further, the influence of sampling period, missing rate and missing type on data repair effect was analyzed:

(1) Sampling period

For randomly missing data (Fig 15), under different data missing rates, the errors of the six repair models all increased with the increase of the sampling period, but the upward trend was moderate, especially after the sampling period exceeded 60 sec, and the moderation trend was obvious. When the sampling period is relatively small, the nearest neighbor interpolation method has the largest error, and the other 5 repair methods have little difference. With the increase of sampling period, the error growth rate of Lagrange interpolation method increases gradually, while that of nearest neighbor interpolation method decreases gradually. For continuously missing data (Fig 16), the data repair effect is similar to that of random missing data, that is, the data repair error increases with the increase of the sampling period, but the upward trend moderates after the sampling period exceeds 10 sec.

Under different data missing rates and types, the data repair effect decreases with the increase of sampling period, indicating that higher data sampling frequency is conducive to data quality control.

(2) Missing rate

By observing Figs 15 and 16, it can be found that the data repair effect of both random missing data and continuous missing data decreases with the increase of data missing rate, but the missing rate has a greater impact on continuous missing data.

(3) Missing type

Under different repair methods, the repair error of random missing data is less than 50 pcu/h, and the repair error of continuous missing data is even as high as 400 pcu/h. However, the multi-section combined repair method based on LSTM proposed in this paper does not increase significantly in the case of continuous missing, and the repair error is always at a low level, which further indicates that the comprehensive repair method is relatively robust.

### Discussion

In this section, data quality control is carried out on traffic flow data, and the accuracy of traffic flow data before and after data quality control is compared. At the same time, the data quality control effect of different repair models is compared, and the influence of sampling period, missing rate and missing type on data repair is analyzed. The experimental results will be analyzed and summarized from the following aspects:

1. With the deepening of data quality control, the accuracy of traffic flow data on each lane has been improved to a certain extent, and the gap between the accuracy of traffic flow data between different lanes has become smaller;
2. Under different sampling periods, missing rates and missing types, the multi-section combined repair method proposed in this paper based on LSTM has the best repair effect;
3. Under different missing rates and types, the data quality control effect of different repair models decreases with the increase of sampling period, indicating that higher data sampling frequency is conducive to data quality control.

The above experimental results prove that the proposed traffic flow data quality control method based on video frame rate and considering cross-sectional geospatial similarity can effectively improve the quality of traffic data. At the same time, the method proposed in this paper only uses the spatial relationship between traffic flows at the road section level to achieve a good control of traffic flow data quality, and has broad application potential in practical traffic management. In this study, although the time characteristics of traffic flow under video frame rate are fully explored, the daily, weekly, monthly and even seasonal changes of traffic flow are not considered. At the same time, the effective combination of multi-source heterogeneous data is also the research direction of traffic flow data quality control. In the follow-up study, different characteristics of multi-source heterogeneous data will be fully utilized, combined with daily, weekly, monthly and even seasonal changes of traffic flow, to further improve the quality of traffic flow data.

At the same time, the traffic flow data quality control under the video frame rate considering the cross-sectional geospatial similarity has achieved good results in the experiment. Based on more accurate traffic flow data, the intelligent transportation system uses technologies such as the Internet of Things, big data and artificial intelligence to interconnect roads, vehicles and traffic management systems to achieve real-time monitoring, intelligent identification and data analysis. This provides people with real-time services such as road traffic conditions, traffic flow prediction, and traffic flow operation risk assessment to improve traffic conditions, reduce traffic congestion, and optimize travel experience. However, the research results of this paper are based on the excellent performance of video frame rate. In practical applications, due to the high cost of the algorithm, there may be some problems such as poor real-time performance. In future studies, we will consider how to achieve similar repair effects at lower sampling frequencies.

## Conclusion

The challenges of traffic flow data quality control are analyzed, and a traffic flow data quality control method under video frame rate considering cross-sectional geospatial similarity is proposed. In the aspect of traffic flow data collection, video-based video frame rate is used to collect traffic flow, speed and density data of multi-section. In terms of data quality control, combining the advantages of lane-level traffic flow data in time dimension and space dimension under video frame rate, the data quality control of traffic flow data is carried out. Firstly, the quality analysis of traffic flow data is carried out to effectively identify missing data and wrong data. Secondly, the quality of traffic flow data is effectively controlled through cross-sectional geospatial similarity analysis, continuous missing preprocessing, piecewise interpolation repair based on cross-sectional geospatial similarity and multi-segment combined repair model based on LSTM.

Experiments were carried out on several road cross-sections to analyze the model performance and the applicability of influencing factors to application scenarios, compare the traffic flow data quality effect, and verify the effectiveness and robustness of the algorithm. The experimental results show that after repairing and optimizing the traffic flow data based on the lane-level traffic flow data quality control method proposed in this paper, the accuracy of each lane is improved to some extent under different experimental conditions. At the same time, the data repair effect decreases with the increase of the sampling period, which proves that higher data sampling frequency is conducive to data quality control.
